# ‘It's an awful thing, but it's better than being dead’: Exploring the emotional circuitry of failed segregation reforms in Canadian prisons

**DOI:** 10.1177/14624745251396554

**Published:** 2025-11-18

**Authors:** Sophie Lachapelle, Jennifer M. Kilty

**Affiliations:** 1 Department of Criminology, 6363 University of Ottawa, Ottawa, Canada

**Keywords:** Segregation, structured intervention units, prison reform, carceral circuitry, emotions

## Abstract

Prison authorities around the world are increasingly adopting more politically palatable alternatives to segregation – often referred to as solitary confinement. In 2019, the Canadian government enacted Bill C-83, legally abolishing segregation in federal prisons – at least, on paper – and established Structured Intervention Units (SIUs) as a supposedly less harmful alternative. Critics have problematized the Correctional Service of Canada's implementation of the SIU model as maintaining previous segregation practices under the rhetoric of ‘reform’. Building upon these criticisms, this article challenges the common refrain that segregation spaces are prisons within prisons, spaces that are somehow different – extraordinary even – in terms of their material conditions and administration. While segregation or SIUs reflect the most austere and punitive form of isolation, they do not exist apart from the wider carceral environment. Developing the notion of emotional circuitry, we examine formerly incarcerated people's (n = 57) experiences of moving between general population, SIUs, and other segregation-like spaces. We highlight the falsity of distinguishing SIUs as a distinct carceral space, and the importance of understanding its spatial, material, and emotional effects as born from the violence of the broader incarceration experience that no degree of segregation-specific reforms can ameliorate.

## Introduction

The disturbing ramifications of segregation – often referred to as *solitary confinement* – are well documented. Insomnia, hallucinations, psychosis, suicidal ideation, depression, anxiety, apathy, hypersensitivity to sensory stimuli, appetite and weight loss, heart palpitations, dizziness, headaches, etc. (e.g. Adamjee, 2022; Andreescu, 2023; Haney, 2018; Kelsall, 2014) are some of the known consequences of carceral isolation. Despite these harms, isolation is a common practice in western prisons, albeit to varying degrees. For example, while the United States holds approximately 122,000 incarcerated men, women, and children (6.5% of the prison population) daily in some form of isolation (Solitary Watch, 2023), England and Wales have a segregation capacity of 1586 cells and their close supervision centres have a capacity of 54 (Shalev and Edgar, 2015). Nearly 10% of England and Wales’ prison population spend at least one night in segregation; of those segregated, 71% spend less than 14 days in segregation, 20% spend between 14 and 42 days, and 9% are segregated for longer than 84 days, while the average stay in close supervision centres is 40 months (Shalev and Edgar, 2015). While Australian territories and jurisdictions use different terminology (e.g. segregation, separate confinement) to describe isolation practices, making it difficult to ascertain how many people are experiencing this form of holding at a given time (Walsh and Blaber, 2023), one study found an average of 1.4% of the Queensland prisoner population was being held in prolonged separate confinement (van den Berg and Krulin, 2019). Regardless of the number of incarcerated people held in segregation, a disturbingly common trend across these western countries – Canada among them – is that Black, Indigenous, and other people of colour are disproportionately subject to isolated confinement. However, it is the mentally, emotionally, and physically torturous nature of segregation (Andreescu, 2023; Batelaan, 2023; Reiter, 2016; Webster, 2015), that is leading prison authorities around the world to consider more politically palatable alternatives (Diorio, 2022; Earle, 2020; Haney, 2018).

In Canada, such legislative changes were catalysed by several high-profile cases of inhumane treatment in segregation (Kilty, 2014, 2018; Parkes, 2017). The most infamous being the death of 19-year-old Ashley Smith, who died on 19 October 2007, in a segregation cell at Grand Valley Institution for Women in Ontario from a self-tied ligature around her neck. Staff filmed her death from the hallway (by law, all cell extractions and use of force incidents are filmed), having been instructed not to enter her cell to cut off the ligature until she fell unconscious. Smith served 4 years in custody – almost entirely in segregation – for an index offence of throwing crab apples at a postal worker as a youth. On 19 December 2013, a coroner's jury returned a verdict of homicide, indicating the inaction of correctional staff contributed to her death, but without a finding of criminal or civil liability. After subsequent inquests and judicial decisions determined that the Correctional Service of Canada's (CSC)^1^ use of segregation violated the *Canadian Charter of Rights and Freedoms*, the Ministry of Public Safety introduced Bill C-83 (An Act to Amend the Corrections and Conditional Release Act and another Act) in October 2018 (Sapers, 2022; SIU-IAP, 2024a).

Prior to Bill C-83, the Corrections and Conditional Release Act (CCRA) provided CSC with the legal means to deploy two forms of segregation: *disciplinary* and *administrative*. Used to enforce institutional rules, disciplinary segregation required an incarcerated person be charged with an institutional violation, receive written notice of said charge, and attend a disciplinary hearing^2^ before placement in segregation for a maximum of 45 days (Sapers, 2022). By contrast, administrative segregation was used to ‘maintain the security of or the safety of any person by not allowing an inmate to associate with other inmates’, and could be enforced for an indeterminate period of time (CCRA, SC 1992, c 20, ss 31(1)^3^; Sapers, 2022). Unsurprisingly, the combination of strict regulations regarding disciplinary segregation and the ambiguous definition of administrative segregation led to reliance on the latter to punish and control incarcerated people (Sapers, 2022).

Becoming official policy in November 2019, Bill C-83 legally abolished disciplinary and administrative segregation and created a supposedly less harmful alternative: Structured Intervention Units (SIUs). Established as stand-alone, multi-level security units in 15 of Canada's 44 federal institutions, SIUs are mandated to achieve three benchmarks that differentiate it from segregation:

1. SIUs are used when there is no reasonable alternative. A review of each SIU placement must be carried out by an Independent External Decision Maker (IEDM^4^) within 30 days, returning the incarcerated person to the general population at the earliest opportunity;
2. SIU residents must receive *a minimum* of 4 hours outside of their cell every day; and
3. SIU residents must receive *a minimum* of 2 hours of meaningful human contact per day (Adamjee, 2022; CSC, 2019).

To ensure SIU implementation met these requirements, the Minister of Public Safety established the Structured Intervention Unit Implementation Advisory Panel (SIU-IAP), which published eight empirical reports on the operationalization of SIUs over its 5-year mandate^5^. Despite consistently reporting that SIUs are different from administrative segregation in name only, neither the Ministry of Public Safety nor CSC has responded with any meaningful policy or operational changes (SIU-IAP, 2024b).

In their final report, the SIU-IAP (2024b) remonstrated ‘the SIU regime has, after 5 years of operation, not accomplished what it was supposed to do, which was to abolish the practice of solitary confinement-like conditions… [and] the situation is not getting better’ (para. 23). For example, Canada's SIU model does not adhere to the UN Special Rapporteur's position that anything more than 15 days in solitary confinement constitutes torture, and that people who are pregnant or are suffering from mental health distress should never be isolated (Méndez, 2011). Instead, there is disturbing continuity in Canada's problematic use of prolonged periods of isolation:

- SIU stays are similar in length, or longer, than they were under the administrative segregation model;
- Indigenous people, Black people, and people with mental health issues have the longest SIU stays;
- Fifty-five percent of people in the SIU do not spend the legislated 4-hour minimum outside of their cell on at least 75% of their days in isolation;
- Half of those in SIUs do not engage in the legislated 2-hour minimum of meaningful human contact during 51% of their days in isolation; and
- Forty-seven percent of all people detained in SIUs between 2022 and 2024 received zero correctional programming while in isolation (SIU-IAP, 2024b).

Furthermore, the SIU-IAP (2024b) cautions that CSC's operationalization of segregation-like practices cannot be fully quantified ‘[t]o the extent that isolating conditions of confinement can be imposed on prisoners anywhere in an institution… these practices largely go unmonitored by any external oversight system’ (para. 5). Solitary-like practices of confinement in non-SIU spaces were confirmed in multiple instances by the SIU-IAP during their institutional visits, at least one IEDM (SIU-IAP, 2024b), and the Office of the Correctional Investigator (OCI)^6^ (2022). Our participants corroborated reports of CSC engaging in segregation-like practices in non-SIU spaces via strategies that obscure their use of extra-judicial isolation. We discuss these strategies in the ensuing analysis.

While the SIU-IAP provided some external oversight of SIUs, they held no mandate to provide oversight for any other penitentiary spaces, which continue to operate with impunity and secrecy. Notably, the SIU-IAP (2024a) reports that ‘many’ incarcerated people request placement in SIUs despite the deleterious effects it has on well-being (Adamjee, 2022; Andreescu, 2023; Haney, 2018; Kelsall, 2014). Rejecting CSC's claim that such requests signal the success of the SIU regime, the SIU-IAP contends they are instead ‘an indication more of the inadequacy of CSC's administration of the rest of the penitentiary’ (SIU-IAP, 2024a: para. 128). Furthermore, neither CSC nor the SIU-IAP includes qualitative data that documents people's material experiences of SIUs in their reports. This article makes a significant contribution by empirically centring the voices of incarcerated people regarding their experiences of the operational shift to SIUs, what Lefebvre (1991) calls representational or lived space, via qualitative data collected through in-depth interviews.

While participants frequently used the same terms – caged, claustrophobic, paranoid, and suicidal – to describe how they felt while in segregation and/or SIUs, several also discussed the *necessity* of isolation in some form or another. Participants perceived segregation as an important option for physical safety and emotional reprieve from the chaos of living among the general population. As David expressed, per this article's title: ‘It's an awful thing, but it's better than being dead’. We contend that maintaining an exclusive analytical focus on the harms of segregation and how to ‘reform’ it obscures the physical, mental, and emotional violence that conditions prison life more broadly and that, for some, necessitates isolation. By considering people's lived experiences of SIUs and hidden spaces of segregation, this article demonstrates that the goals of the SIU reform do not align with their materiality and thus that the promises of this model have not been met.

After contextualizing our analysis in the carceral and emotional geographies literature, we describe the methods used for our project (*Feeling the Carceral*). Next, we develop Gill et al.'s (2018) notion of *carceral circuitry* – which ‘emphasizes the spatial and temporal rhythms of mobility within carceral settings… the periodicity, frequency, and pace of carceral cycles’ (189) – to include emotions. Conceptualizing the idea of *emotional circuitry*, we challenge the common refrain that segregation spaces are prisons within prisons, spaces that are somehow different – extraordinary even – in terms of their material conditions and administration. While SIUs may reflect the most austere form of holding, they do not exist apart from the wider carceral environment. By examining formerly incarcerated people's emotionally circuitous experiences between general population and isolated spaces, we highlight the falsity of distinguishing segregation as a unique carceral space, and the importance of understanding its spatial, material, and emotional effects as born from the broader incarceration experience.

Introducing emotions into Gill et al.'s (2018) model of carceral circuitry in the context of SIUs also helps problematize established approaches to studying carceral rhythms^7^. While prison spaces are often discussed in terms of temporal and spatial predictability (e.g. yard time, mealtimes, sequentially gated corridors, curfews, cell counts, etc.) (Heaney, 2023; Herrity, 2024), placement in a SIU or in segregation-like conditions interrupts carceral routines and often does so suddenly following a highly charged incident. Largely unable to predict the rhythm of SIU placements (i.e. for whom, when, or where SIU placement will occur) yet confident that such events are inevitable in prison, participants discussed an affective atmosphere demarcated by fear and dread as they walked on eggshells to avoid isolation. For participants, the inability to predict the rhythm of the circuit between general population and SIUs contributed to the emotional freneticism experienced in carceral spaces more broadly. This reframing usefully illustrates the continuity of emotional harms across carceral spaces, thus problematizing the very notion of segregation reform.

### Carceral and emotional geographies

Following Foucault's (1975/1977) explanation of modern society's ‘subtle, but graduated carceral net’, (297), carceral geography tracks the movement of carceral logics both in- and outside of prison, problematizing the idea of a fixed boundary between the two (e.g. Gacek, 2022; Gilmore, 2007; Jewkes and Laws, 2021; Moran et al., 2017, 2018; Smoyer and Blankenship, 2014; Turner, 2020) by exploring the mobility of people (Brooks and Best, 2021; Garneau and Lehalle, 2021; Russell et al., 2022), spatial practices (Kilty and DeVellis, 2010), labour (Richardson and Thieme, 2020), objects (Baer, 2005), and ideas (Moran and Jewkes, 2014) beyond prison walls. Whether examining hyper-carceral and policed spaces of inner-city communities (Brown, 2014) and police custody (Wooff and Skinns, 2018), *transcarceral* parole and probation spaces (Kilty and DeVellis, 2010; Welsh, 2019), or *quasi-carceral* spaces like prison visiting rooms (Moran, 2013), carceral geographers demonstrate the diffusion of carceral practices to discipline, control, and punish outside the physical confines of the prison. As such, carceral geography explores the notion of carcerality not as a defined set of spatial characteristics, but rather as a *practice* of power (Foucault, 1975/1977) that is spatially mediated and produced (Lefebvre, 1991).

Understanding carcerality as a spatially mediated practice is a useful frame for studying Canada's segregation reform. As many SIUs are located in old segregation units, the new regime was not introduced to change the physical make-up of Canada's federal segregation spaces, but rather ‘to abolish the *practice* of solitary confinement-like conditions’ (SIU-IAP, 2024b: para. 23; emphasis added). To evaluate the relative success or failure of the SIU regime, we explore the degree to which solitary confinement-like *practices* continue to exist in Canadian federal prisons – in policy, practice, and perhaps most importantly, at the level of *experience*, or rather, representational space (Lefebvre, 1991).

To this end, carceral geographers have begun exploring prison as a unique emotional landscape (Crawley, 2004; Liebling, 2013) to better understand the relationships between people, objects, and ideas as they move within and across carceral spaces (Lachapelle and Kilty, 2023a, 2023b; Crewe, 2015; Crewe et al., 2014; Gacek, 2018; Herrity, 2024; Jewkes and Laws, 2021; Walby and Cole, 2019). Analysing carceral emotional geographies necessitates disrupting Cartesian mind/body dualisms (Ahmed, 2014; Brennan, 2004) and working ‘to understand emotion – experientially and conceptually – in terms of its socio-*spatial* mediation and articulation rather than as entirely interiorised subjective mental states’ (Bondi et al., 2005: 3). As such, we take up Gould's (2009) definition of emotion as a ‘personal expression of what one is feeling in a given moment… [which] brings a vague bodily intensity or sensation into the realm of cultural meanings and normativity, systems of signification that structure our very feelings’ (20, 21). This analytical embrace of emotions is a welcome one as it is often through emotional experiences of a place that we evaluate it as having a unique culture or quality (Herrity et al., 2021; Herrity 2024), with the material body becoming a vehicle through which emotions are felt and expressed in relation to carceral power (Kilty, 2014, 2018; Moran, 2014).

This emotional turn has inspired scholars to develop a thorough understanding of the ways in which modern understandings of punishment are, in fact, ‘founded in emotion’ (Brown, 2002: 417). Indeed, prison and state authorities readily use emotion management techniques – including prison reform initiatives – to foster specific emotions (e.g. anger, disgust, pity, compassion, etc.) that contribute to the maintenance of hegemonic, but dissonant narratives of prison as a moral institution that is simultaneously retributive *and* humane (Bogosavljević and Kilty, 2024; Canton, 2015; Carvalho and Chamberlen, 2018; Freiberg, 2001). Building on this literature, which mobilizes emotion to better understand ‘what work does prison do? for whom? and to what end?’ (Gilmore, 2002: 15), more recent scholarship in carceral geography has tried to map these emotionally charged social narratives of punishment onto and within incarcerated people's embodied, material, and spatially mediated experiences of prison (Liebling, 2011).

Although prison is often depicted in popular media as characterized by feelings of fear or mistrust, scholars are beginning to challenge such monolithic characterizations, which fail to ‘describe the way in which prisons have a distinctive kind of emotional geography, with zones in which certain kinds of feelings and emotional displays are more or less possible to experience and exhibit’ (Crewe et al., 2014: 57). For example, while many incarcerated people characterize prison as an aggressive, violent, and ‘macho’ space (Crewe et al., 2014: 63; Laws, 2019), others have shown that certain correctional spaces allow for personal growth, the development of intimate relationships, or emotional catharsis (Lachapelle and Kilty, 2023a; Gacek, 2018; Hjørnevik and Waage, 2019). Cells can be both a comforting, homelike space and an unbearable reminder of being unable to go home (Baer, 2005; Chamberlen, 2016); visiting rooms are places of relief, joy, and intimacy at the same time as they are haunted by painful memories of trauma and forced farewells (Aiello and McCorkel, 2018; Moran, 2013; Moran and Disney, 2018, 2019); and spaces of admissions, discharge, and transport are characterized by both extreme boredom and intense fear (Lachapelle and Kilty, 2023b; Eife and Kirk, 2021; Garneau and Lehalle. 2021). Even experiences in spaces of extreme isolation – as we discuss in detail below – are emotionally heterogeneous (Gonzalez, 2025; Laws, 2021). By mapping the material and relational make-ups of different prison spaces, carceral emotional geographers have highlighted the acute emotional whiplash incarcerated people experience while imprisoned (Laws, 2022). However, emotional geographies of prison have, thus far, been limited to the domestic, recreational, and therapeutic spaces of correctional institutions, ultimately excluding spaces that do not fit within this ‘emotional map’ (Jewkes and Laws, 2021: 396). Furthermore, several geographers have applied an emotions lens to discrete carceral spaces (Brooks and Best, 2021; Russell et al., 2022), few have done so to explore circuits *within* them. Yet, the borders between prison spaces are not only ‘edges [but] are also interfaces… while borders highlight the distinction between places, they also connect places into relationships with each other and with non-contiguous places’ (Gilmore, 2007: 11). Preoccupied with theorizing the prison boundary ‘as a messy, blurry, irregular entity’ (Turner, 2020: 44), carceral geography has ignored how the emotions felt in seemingly distinct prison spaces inform, maintain, resist, and mask each other, and what the relationships between these spaces reveal about the operationalization of carceral power. This paper helps remedy this gap by analysing the emotional circuitry between spaces designated for isolation, spaces that have become solitary confinement-like through quotidian spatial practices (Lefebvre, 1991), and the rest of the prison. As such, we do not discuss specific emotions experienced by incarcerated people in isolation, but rather focus our analysis on the ways in which emotions are taken up in spatially mediated – namely, *circuitous* – ways that maintain carceral power.

### Emotional circuitry of Structured Intervention Units

Attending to the *emotional circuitry* of SIUs demonstrates the inextricability of isolated spaces from the rest of the prison. In other words, if we truly wish to ameliorate the material and psychological violence of segregation, we must consider its place and function in the prison's broader emotional geography. Adopting a circuitry lens helps identify problematic patterns that are otherwise obfuscated by neoliberal discourses of reform that, according to Gill et al. (2018), point

towards the importance of recurrence… Inherent to this approach is the recognition that circuits return to their own starting point… [A circuitry lens] connects places that are ‘typically encountered as discrete’ but that have been caught up in the same processes of marginalization and exclusion (189, 194)

Circuits are a network or series of interconnected activities, events, people, or places frequented by a particular group. While Gill et al. (2018) contend that there are three kinds of circuits in carceral spaces – people, objects, and practices – this model fails to consider emotions as important components of carceral circuits or as constituent elements to the operationalization of carceral power.

Following Gill et al.'s (2018) argument that circuits return to their starting point, the most obvious circuit between SIUs and the rest of the prison involves the movement of incarcerated people; anyone who is placed in solitary confinement has come from the general population and will – eventually – return there. Incarcerated people are isolated for many reasons, including: punishment for breaking institutional rules, ‘acting out’, or refusing to cooperate with correctional staff; physical protection; emotional reprieve from the broader prison environment or due to a lack of mental health support during a crisis. While analysing this carceral circuit may provide rich experiential data about these spaces, such a study risks the continued pathologization of incarcerated people's emotional responses to structural violence, unintentionally eliding the undercurrents of carceral power that give rise to the perceived necessity of solitary confinement-like spaces (Tuck and Yang, 2014). Conversely, attending to the *emotional circuitry* of SIUs allows us to acknowledge incarcerated people's experiences of pain and humiliation while examining circuits of carceral power that move across seemingly discrete spaces. This dovetails with Lefebvre's (1991) position that space is both a product of and a shaping force in our lives and can be used to exert power, reinforcing dominant structures and systems that marginalize certain groups.

Our notion of emotional circuitry acknowledges the emotions that people feel in specific carceral spaces and considers the conditions that give rise to them and how institutional actors, systems, and discourses *respond to* these emotions in spatially mediated ways (i.e. isolation). Shifting one's analytic focus away from the individual circumstances that result in segregation placements (e.g. rule breaking, resisting authority, antagonistic relations with staff and/or incarcerated people, the need for physical protection and/or emotional reprieve, mental health crises, etc.) and towards the emotions permeating these placements (e.g. fear, anger, frustration, despair, anxiety, confusion, overwhelm) helps reveal how the prison environment produces violent and unbearable social conditions that surface particular feelings and emotional responses. In other words, an emotional circuitry lens acknowledges that ‘emotion is a constant companion of power’ (Layder, 2004: 17), where conditions of confinement produce a frenetic emotional restlessness that moves with people through carceral spaces, building in intensity until, for some, it culminates in forms of extreme isolation.

The SIU emotion circuit has temporal signifiers denoted by policy – that residents should receive 4 hours out of cell and 2 hours of meaningful contact per day, be released at the earliest possibility, and have an external case review every 30 days – that have not materialized in practice (|SIU-IAP, 2024a, 2024b; Sapers, 2022). Moreover, any agency and emotional engagement a person may feel in requesting transfer to a SIU is countered by their inability to determine when they leave and are no longer subject to this spatial practice, creating an arrhythmia in the circuit. The momentary relief one might experience from the emotional intensity of prison life when isolated is also accompanied by more acute psychological effects that worsen over time (Adamjee, 2022; Andreescu, 2023; Haney, 2018; Kelsall, 2014). When the individual moves back into the general population, they bring those experiences of psychological distress with them, perpetuating an emotional atmosphere marked by aggravation and exasperation.

Given the conditions of confinement in Canadian prisons, solitary spaces may be requested by those who require ‘alone time’ to cope with the terror of incarceration. This does not mean that we position segregation, in either its former or current incarnations, as a positive carceral management strategy. Rather, we contend that requests to be isolated enlighten us to the emotional distress born from the general conditions of confinement, which – as demonstrated below – are the starting point of this emotional circuit.

## Methodology

This paper is part of a large qualitative research project entitled *Feeling the Carceral*, designed to examine the emotional terrain of federal penitentiaries in Canada. The main objective was to develop thick, rich descriptions of carceral emotional geographies, including what emotions are experienced in specific spaces and the socio-political, cultural, interpersonal, and spatial processes that regulate emotional expression. This included examining how power circulates within carceral spaces, shaping the emotional expression of both staff and incarcerated people. The interview guide was designed to explore the emotions participants felt in different carceral spaces, including admissions and discharge; cells, ranges, or houses^8^; programming and educational spaces; medical units; segregation and SIUs; common spaces; and various reintegration spaces. Using open-ended prompts, participants were asked to describe the material make-up and content of each physical space and to narrate both routine and more unique incidents they experienced in each space.

The research team conducted N = 57^9^ (20 women, 37 men) semi-structured interviews with formerly federally incarcerated people recruited through non-profit community-based organizations. Following approval from our university's Research Ethics Board and the Executive Directors of these organizations, staff were asked to distribute recruitment materials to service users, who contacted one of three doctoral-level research assistants by phone or email to indicate their willingness to participate and to schedule an interview. On the day of the interview, after reading through the consent form with the participant, interviewers secured oral consent before commencing. Interviews were conducted virtually due to the COVID-19-related public health guidelines that were in place between December 2020 and August 2021 and lasted 90 min on average. Considering SIUs were introduced in 2019, some participants spent time in SIUs, in both SIUs and segregation, or in segregation but not SIUs. To underscore the credibility of participant narratives, we cite only those who spent time in a SIU, were inmate committee^10^ members who visited and heard complaints from people in SIUs, and/or those who experienced ‘hidden’ segregation cells following the implementation of the SIU regime (SIU-IAP, 2024a, 2024b). To protect anonymity, when using a direct quote we cite the participant's pseudonym.

Following verbatim transcription, the interview transcripts were analysed using NVivo qualitative analysis software via a continuous process of thematic narrative coding (Riessman, 2008) to understand how different carceral spaces elicit emotions, feelings, and sensory memories. Thematic narrative analysis concentrates on ‘what’ is said rather than how a speech act is organized. Per narrative inquiry methods we coded large swaths of text to keep stories intact and paid careful attention to the different ways that participants described feelings and memories they had in different carceral spaces (Riessman, 2008). This meant coding excerpts with multiple codes that were cross-referenced to understand the connections between certain emotions and spaces across participants.

Participants’ experiences of isolation in SIUs mirror those that have been long-documented in the existing literature, including feeling abandoned, dehumanized, angry, distressed, and unsupported by CSC (Kilty, 2014, 2018; Kelsall, 2014; Webster, 2015). Despite participants’ universal agreement that isolation is harmful and increases the depth, weight, and tightness (Crewe, 2015) of one's carceral experiences, several participants also noted that some people request a transfer to a SIU. Such requests reveal how the emotional circuitry of SIUs is directly linked to the harms of carceral spaces *in toto*. We explore participants’ experiential accounts, connecting the emotional circuitry of SIUs to the prison as a total institution (Goffman, 1963) to illustrate how segregation reform problematically legitimates incarceration despite the emotional harms these spaces engender.

### Analysis

It's inhumane, but it's not new

Given repeated SIU-IAP reports that SIUs bear little difference from administrative segregation, it was unsurprising that participants described their feelings while in isolation – both before and after the introduction of Bill C-83 – in similar ways:

This is where someone would say we want to call it a “step-down” unit because it doesn’t sound as bad as segregation. Call it whatever you want, but it's still what it is. (David)

One hundred percent it's a show pony, you know what I mean? [Staff] just sit there saying “this is what we’ve done to combat segregation,” but there's all these lawsuits going on. It's inhumane, but it's not new. (George)

Lynn, who was federally incarcerated exclusively under the SIU regime, still referred to it as ‘seg’ when discussing how isolation was used to punish mental distress.

There was quite a period of time where I felt like giving up. I became suicidal, I was self-harming. Trying to conceal that only made my situation worse. It made me really nervous about people seeing it and putting me into seg. With suicide watch, you get put into seg. Everything is taken away from you and I was just so scared. I was scared of being honest about having mental health issues… that's a lot of the issue, we’re not addressing it because we’re fucking scared, because we could potentially be put into seg or be sent to some institution and taken away from friends that we’ve made. All these things can happen, and it feels like a punishment for having mental health issues… You’re not completely safe in that space, you still have to worry about what you’re saying, which is hard. You’re scared and paranoid and just on edge all the time. (Lynn)

While Lynn was clearly afraid of being placed in the SIU, the fear and anxiety of said placement also reveal the extreme level of emotional distress she endured in the general population. Feeling suicidal amidst a lack of institutional mental health support and being acutely aware of CSC's policy to forcefully remove people experiencing suicidal ideation to the SIU – Lynn lived in a constant state of paranoia, unable to trust correctional staff would act in her best interest.

That said, many participants – even some who experienced unmitigated horrors in older segregation units, SIUs, or both – suggested that some form of segregation was integral to the smooth, day-to-day operation of prisons:

CSC does need [segregation] in some form. When you have someone who lost a loved one and maybe can’t get out or is going out in a few days and they want some quiet time, that's where they want to go. If you have someone who gets denied parole, sometimes that's where they want to go. If you have someone that has a drug debt or any debt in the institution and for their safety, that's where they want to go… If someone's having a breakdown and there's no mental health staff, or even healthcare staff, around to help them, it's up to [other incarcerated people] to babysit them, and [prison guards] have as much knowledge as we do. That's not a very good structure. People have to stay in these units until they get a chance to get somewhere safe. I know it's not the best. It's an awful thing, but it's better than being dead… And I know what the public says, and I know the outcry, but take it from the guys who have done the time, you can’t eliminate such things. You can be smarter about it. I’m not saying it's okay to be locked in segregation for months and years. Let's be realistic here, but I’m saying sometimes there are people that need to go there. (David)

It is important to situate David's critique within the context of an understaffed and poorly resourced institution, where his ability to envision a more rehabilitative approach was limited by the immediate emotional needs of his incarcerated fellows. By incorporating emotions into Gill et al.'s (2018) concept of carceral circuitry, we can begin to see how David's claim that some form of segregation is required is part of a deceptively violent, but emotionally distinct circuit. As Gill et al. (2018) state:

by “following” phenomena [like emotions], the complexity, contradictions and over-arching logics of their journeys can be laid bare in a way that is not possible if only particular stages or places… are examined in isolation… Such an ontology helps to undo the unhelpful “boxing-up” that a compartmentalized world view can promote, which makes it hard to understand the entanglements between… carceral spaces. (186).

Rather than justifying segregation as integral to an efficient prison or interventionist agenda, David's narration of the emotional function of extreme isolation within a broader context of violent institutional neglect reveals how requesting a transfer to an SIU indicates the kind of visceral emotional fracturing – and complete lack of care – incarcerated people experience daily.

For participants like David, SIUs serve two emotional functions: first, as a space to experience emotional reprieve and quiet that could not be accessed elsewhere; and second, to keep those who are unable to regulate their emotions safe from harming themselves or others. It is important to note that participants who discussed the SIU as a place of emotional reprieve always did so in comparison to the harms of the broader prison environment, showcasing the contours of this emotional circuit:

I’m OK with my own company. I can read six or seven hours of the day, read a book without any problem. Your headaches are diminished greatly because you’re not out dealing with the politics of whatever's going on. You don’t really have to deal with staff a whole lot… And then you go back out into the general population and all the bullshit starts again. (Drew)

I’ve probably spent four or five years in segregation… At first it freaked me out… But I spent so much time in the hole, that I didn’t mind being isolated after a while… there were so many people were getting stabbed, there were so many fights there and so many incidents of people fighting with guards, we were always locked down… It's better to be in the hole during a lockdown because you get more yard. I always knew that I could go to the hole and just relax and chill out and calm down. (Ryan)

As these quotes reveal, even in cases where participants welcomed the SIU, it was because living conditions elsewhere felt unbearable. Fear of physical violence, sensorial overwhelm (Lachapelle and Kilty, 2023a; Herrity, 2024), and material deprivation were so rampant that participants like Drew and Ryan *preferred* the torturous environment of ‘the hole’ despite knowing the mental, emotional, and physical consequences of isolation. Drew's statement also reveals how agonistic relations with staff contribute to incarcerated people seeking increased isolation and confirms SIU-IAP (2024a, 2024b) findings that SIU residents are not receiving the mandated requirements of 2 hours of meaningful contact and 4 hours out of cell per day. Situating incarcerated people's motivations for requesting transfer to an SIU within the prison's broader emotional geography illuminates that common spaces and the relations within them are the starting point of this emotional circuit, subsequently problematizing segregation *reform* as an effective solution to carceral violence.

Similarly, an emotional circuitry lens calls into question the correctional claim that segregation prevents emotionally distressed people from experiencing further harm. Not only does isolation exacerbate mental distress and violence, but as Reiter (2016) contends,

[p]risoners struggle to work their way out of [segregation]. And then they struggle to survive the sensory overload and chaos outside the box. Their stories reveal how supermaxes have failed to distinguish violent from nonviolent prisoners, failed to distinguish mad from bad, failed to cure violence, and failed to meet a battery of basic human needs. (194)

This framework is ‘analytically useful because it signifies not only the continuous and coerced movement of bodies through carceral systems and spaces, but the transformation or production of ‘criminal’ and carceral subjectivities’ (Russell et al., 2022: 156). While this transformation is operationalized through spatial practices (Reiter, 2016), Lefebvre (1991) emphasizes that representations of space – how professionals design and imagine space – produce these subjectivities, in this case those that are deigned dangerous, risky, and at risk. Indeed, participants detailed their transformation into a criminal subject as they moved between general population and SIUs:

Anytime I would get out, it would only be for a couple of days before I’d go straight back to seg. It was just that routine. I was just like, fuck it, I’m already in trouble anyway, what are you going to do? They’ll just do the same thing. Pepper spray you, strap you to your bed… I was like a completely normal person. And then after a while, I just start being fucked up. I just wanted to hurt myself, smashing my head off the fucking wall just going crazy in there. When I first got to max, my friend died, we used to chill all the time. And when I came out of max, they tried to put me in her cell, and I fucking lost it, and they wouldn’t put me in another cell. And that was kind of the start of why I went to seg. And then it was just always fucking something. They end up trying to put me in that same room that she fucking killed herself in, like not even a couple of weeks after she fucking died. So, they would just dry cell^11^ me. Most of the time I was in there, I had completely nothing, just fucking bored. No paper, no book, no nothing. Just in a smock with a guard in front of you and a camera. (Dyani)

Cycling in and out of the SIU is not only emotionally, mentally, and physically jarring, but this circuit also forms part of a larger system designed to contain and manage risk (Gill et al., 2018). In such degrading environments, the creation of criminal subjectivities born from an inability to regulate ‘angst, irritability, and aggressive outbursts [are] almost inevitable’ (Walker et al., 2020: 885). Indeed, while ‘[t]he old logistics of carceral geographies and mobilities may be shifting toward reworked scripts – reforms that are disrupting the geography of prisons without unearthing their logistical’ or ideological ‘frames’, such as the creation of SIUs, ‘are not the crux of the “problem”’ (Brooks and Best, 2021: 472). They are simply where the problem is most visible.

*Segregation is basically just another unit now*

Prison watchdogs expressed concern that Bill C-83 created legislative loopholes in preventing solitary confinement-like practices. The SIU-IAP (2024b) and the OCI (2022) both contend that CSC's establishment of isolation ranges – deemed *therapeutic units* – since the implementation of SIUs are an attempt to keep SIU numbers low and evade media attention:

A wide range of restrictive confinement conditions and practices exist outside of SIUs that are subject to little or no external oversight or independent monitoring… At one institution, out-of-cell time and conditions of confinement in the regular units and the Therapeutic Range was consistently poorer and below the standards prevailing in the SIU. Indeed, *outside the SIU, the rest of the institution operated as if it were a former administrative segregation range*, with fewer than three hours of out-of-cell time. At another institution, the pressure to keep SIU numbers low and an increase in incompatible sub-populations resulted in the use of “hidden cells” (a term often used by staff at this facility), where prisoners are kept in solitary confinement-like conditions for weeks on end. (OCI, 2022: 78, 81; emphasis added)

Reiter (2016) contends that this phenomenon arises due to both definitional and interpretative problems, where prison officials consider any ‘privileges’ like access to television, proof that the person is not in solitary confinement. FTC participants confirmed solitary confinement-like practices operating outside of designated SIU spaces. For example, Miguel and Ryan were incarcerated in different institutions across the country from one another, but both described disturbingly similar instances of staff enforcing conditions of extreme isolation outside of the SIU and its legislated accountability measures:

They implemented the new regulations with a cap. They’re not allowed to put people seg anymore. [When] they stopped doing that, they put people in like cell restriction so you can get locked in your cell. (Miguel)

The guards are so mean, the institution is so fucked up that they took segregation away… they put you in a unit now where you’re not segregated to your cell, right? They’ll keep you in the cell if you act up. If you chill out, they’ll let you out in a little calm area with a bunch of guards, but you’re still segregated. Segregation is basically just another unit now… you’re basically stuck in your cell all day. (Ryan)

Given the presence of solitary confinement-like conditions on non-SIU ranges, at least one IEDM, the SIU-IAP (2024b), and the OCI (2022) – as well as our participants – have reported that, for some, SIUs can be preferable to other living units. While the SIU model is just as inhumane as its predecessor (SIU-IAP, 2024a, 2024b), it also paradoxically compounded the unbearable social conditions in other prison spaces, leaving incarcerated people living in the general population without the legislated protections against, and external oversight of, the solitary confinement-like conditions to which they are subject outside of SIUs.

Perhaps even more disturbing is that this is not the only way that CSC has manipulated SIU reporting to avoid further public scrutiny. CSC also performs involuntary transfers of incarcerated people to different institutions – sometimes across the country – to keep SIU placement numbers low (SIU-IAP, 2024b). George described the impact of these transfers as ‘emotional terrorism’:

Let's say I’m at the minimum [security institution] and I do something. Instead of sending me to a Structured Intervention Unit, having to transfer me, having to fill out paperwork, they’ll just emergency involuntary transfer me and raise my security classification. Who do you write your rebuttal to on that? You write it to the Warden, the same guy that signed off on your emergency involuntary transfer. It makes no sense and it's such a loophole… I’ve seen this happen countless times… By the time they’re at the point that they want to send you to an SIU, they already do not like you… It's *emotional terrorism* and it really has a very significant negative emotional impact on whoever's in segregation.

George's experience of involuntary transfers to a higher security institution illustrates how representations of space are designed to exert power and reinforce existing systems and structures (Lefebvre, 1991). Rather than alleviating the emotional trauma of segregation, the SIU model created operational loopholes wherein correctional staff employ policies and spatial practices (Lefebvre, 1991) that prolong and intensify incarcerated people's experiences of isolation and ensure movement through the frenetic emotional circuit running between isolation spaces and the rest of the prison. SIUs have not enabled the abolition of segregation in Canadian penitentiaries; on the contrary, this regime further entrenched the spatial practice of extreme isolation to manage the emotional responses of incarcerated people as they are exposed to violent conditions of confinement that are hidden from public scrutiny.

## Conclusion

Paying attention to the insidious operational similarities between two ostensibly different policy regimes – segregation and SIUs – is gravely important. While it is not an exaggeration to suggest that incarcerated people's lives depend on the continued scrutiny of independent investigations of prison policy development and implementation (Kilty, 2014, 2018; Parkes, 2017), the investigatory emphasis on segregation reform comes at the cost of ignoring the less visible forms of violence that incarceration practices facilitate. That some incarcerated people request a transfer to a SIU despite their brutal material conditions is precisely why we cannot compartmentalize spaces of isolation from the wider prison environment. Doing so only obscures the full circuit of carceral violence and confuses attempts to challenge the existing structures that gave rise to carceral harms in the first place (Parkes, 2017; Russell et al., 2022). Considering the *emotional circuitry* of SIUs illustrates how incarcerated people's experiences of segregation are mediated by the emotional environment of the broader prison (Kilty, 2018; Brooks and Best, 2021). Instead, we advocate for interventions that are decidedly abolitionist, anti-carceral, and structural in nature. Beginning with the experiential requires analytically centring the voices of incarcerated people, as policies drafted without consulting those affected by them are more likely to maintain oppressive, exclusionary structures of power.

Merging an emotions lens with the notion of circuitry (Gill et al., 2018) and Lefebvre's (1991) theoretical insights on the social production of space provides a unique framework for understanding that spaces are not neutral settings, but rather complex socio-political arenas where emotions, power, and everyday practices intersect to produce and shape subjectivity. By attending to the emotional dimension of the circuit that connects isolation and collective prison spaces by way of the material experiences expressed by formerly incarcerated people, we have shown that Canada's SIUs, like segregation units before them, are not discrete prison spaces. Rather, they are spatial practices that produce and shape emotions in ways that reveal how carceral power marks isolated subjects as inherently at risk, risky, or dangerous. Reform efforts that continue to uphold the false notion that segregated spaces are necessary and distinct – prisons within prisons (Haney, 2018) – does not alter the production of such criminal subjectivities, it only reinforces them (Reiter, 2016). Such reforms fail to understand that the origins of troubled feelings and emotions commence in collective spaces, travel with the individual as they move to/from a segregated space, and are amplified by isolation.

In their efforts to suggest SIUs are a successful alternative to – and thus qualitatively different from – segregation units, CSC manipulated their representations of SIUs (Lefebvre, 1991) by way of faulty usage reporting (Sapers, 2022; |SIU-IAP, 2024a, 2024b). To keep SIU usage rates low, CSC invoked certain spatial practices (e.g. institutional transfers, transfers to higher security units, hidden cells) that have subsequently transformed non-SIU spaces into hitherto undisclosed and thus unreported segregation units that are not subject to external oversight. To step outside the inherently ill-fated reform cycles that further entrench rather than resist carceral structures (Brown, 2014; Parkes, 2017), we advocate for a more transformative approach to justice that would decarcerate and liberate people from such inhumane spatial practices.

Reforms have failed to confront the fundamental principle underlying [segregation spaces]: the assumption that such maximally restrictive institutions are either necessary or useful. The isolation of any subset of allegedly dangerous prisoners, in excessively restrictive and fundamentally invisible institutions, enables a special zone of bureaucratic discretion that is not only hard to see but also impossible to regulate. Real reform must question sweeping claims about dangerous prisoners and render concrete walls transparent—making democratic oversight possible again. When that happens, people might decide whether we ever wanted, or needed, these institutions in the first place. (Reiter, 2016: 205)

While we do not have the space to outline a vision for this imagining herein, we maintain that attending to emotions in analyses of carceral reforms, interventions, and practice is essential for understanding their impact, success, or failure. For without this lens, we would not have seen the ways in which SIU reform rhetoric obscures the harms of incarceration more broadly.

## Notes

1. CSC is the government agency charged with the administration of federal penitentiaries in Canada.

2. Disciplinary hearings are internal, meaning incarcerated people do not have legal representation.

3. Retrieved from: https://laws-lois.justice.gc.ca/eng/acts/c-44.6/20160617/P1TT3xt3.html

4. IEDMs are appointed by the Minister of Public Safety to review and reassess SIU placements, including with respect to their duration, frequency, conditions of confinement, and health care provision. The SIU-IAP (2024a, 2024b; Sapers, 2022) has repeatedly problematised the lack of transparency surrounding the IEDM appointment process, calling into question whether they can provide true external oversight.

5. The Minister of Public Safety appointed Howard Sapers – the former Correctional Investigator of Canada (2004–2016) – as the Chair of the SIU-IAP in 2021. An additional eight members were selected by the Ministry of Public Safety, in consultation with Sapers, and included current and former public service employees, criminologists and forensic experts, as well as members of the public known for their advocacy for incarcerated peoples’ rights both in and out of prison. The SIU-IAP was provided administrative support by the Ministry of Public Safety, but independently determined its own work plan, methodology, and which individuals/institutions to visit as part of its mandate. It should be noted that none of the panel members identified as having lived experience of incarceration (Sapers, 2022). The SIU-IAP's mandate ended in December 2024. Their reports are available online: https://www.publicsafety.gc.ca/cnt/cntrng-crm/crrctns/siuiap-ccuis-en.aspx

6. The OCI is the ombudsman for federally incarcerated people. In addition to producing an annual report on the state of federal corrections, it receives, investigates, and resolves complaints and grievances submitted by incarcerated people. CSC is not legally required to implement the OCI's annual recommendations (*CCRA* SC 199*2*, c 20).

7. Lefebvre's (1991) ‘rhythmanalysis’ suggests being immersed in a space is the best way to understand the temporality of different circuits; unfortunately, we, like most scholars, were prohibited access to Canadian prisons for research.

8. Minimum security institutions have communal ‘house’ or ‘cottage’ style buildings where incarcerated people share common spaces and cook for themselves.

9. When asked about race, participants self-identified as: 63% white; 23% Indigenous; 3.5% Black; and 8.7% other persons of colour.

10. Inmate Committees enable select groups of incarcerated people to provide input to CSC management on institutional operations, less those relating to security.

11. A cell without plumbing so that staff can monitor the individual while awaiting the expulsion of ingested contraband.
